# Studies of indirect and direct effects of hypervitaminosis A on rat bone by comparing free access to food and pair-feeding

**DOI:** 10.1080/03009734.2018.1448020

**Published:** 2018-06-24

**Authors:** Thomas Lind, P. Monica Lind, Lijuan Hu, Håkan Melhus

**Affiliations:** a Department of Medical Sciences, Section of Clinical Pharmacology, Uppsala University, Uppsala, Sweden; b Department of Medical Sciences, Section of Occupational and Environmental Medicine, Uppsala University, Uppsala, Sweden

**Keywords:** Bone, bone turnover serum biomarkers, pair-fed, pQCT, rat, vitamin A

## Abstract

**Background:**

The most prominent features of hypervitaminosis A in rats are spontaneous fractures and anorexia. Since caloric restriction induces alterations in bone, some effects could be secondary to loss of appetite. To clarify the mechanisms behind vitamin A-induced bone fragility it is necessary to distinguish between direct and indirect effects.

**Materials and methods:**

In this study we compared rats fed high doses of vitamin A both with pair-fed controls, which were fed the same amount of chow as that consumed by the vitamin A group to keep food intake the same, and to controls with free access to food.

**Results:**

In contrast to the pair-fed animals, rats in the free access group fed high doses of vitamin A for 7 days had 13% lower food intake, 15% lower body weight, and 2.7% shorter femurs compared with controls. In addition, serum biomarkers of bone turnover were reduced. Peripheral quantitative computed tomography of the femurs showed that the bone mineral content, cross sectional area, and periosteal circumference were similarly reduced in the pair-fed and free access groups. However, bone mineral density (BMD) and cortical parameters were only significantly decreased in the free access group.

**Conclusions:**

Our data indicate that the major direct short-term effect of high doses of vitamin A on rat bone is a reduced bone diameter, whereas the effects on bone length, serum biomarkers of bone turnover, BMD, and bone cortex appear to be mainly indirect, caused by a systemic toxicity with loss of appetite, reduced food intake, and general effects on growth.

## Introduction

The first observations of hypervitaminosis A were published in 1925 by Takahashi et al. (1). The symptoms of rats and mice were ‘alopecia of the head, which develops first of all, and then paralysis of the hind legs and at last they will not be able to stand. All this while serious emaciation is gradually developing’. Using X-ray examination, two independent investigators (2,3) revealed in 1933 that this ‘paralysis of the hind legs’ was caused by spontaneous fractures of the long bones. Strauss (4) reported a thinning of the long bones, especially at the diaphysis, with a very thin cortex in young rats, which could explain the fractures. In 1945 the toxicity of high doses of vitamin A was proven beyond doubt when it was possible to use purified, crystalline retinyl acetate (5), and the most characteristic lesions were skeletal fractures and hemorrhage. Rodahl (6) used systematic X-ray examinations at various stages of hypervitaminosis A and found that there was a gradual reduction of the diameter of the femur and an abnormal thinning of the cortex. By the mid-1950s there were 31 different studies of vitamin A toxicity reporting skeletal lesions (7). More recent investigations have confirmed and extended these early observations (8–10).

It is well-known that vitamin A toxicity is associated with reduced food intake (11). Since caloric restriction also induces major alterations in bone phenotype (12–15), several of the bone changes reported in the literature may be a consequence of indirect effects on appetite and general growth rather than direct effects of vitamin A. By comparing pair-fed animals, i.e. using controls fed the same amount of chow as that consumed by animals fed high doses of vitamin A, with animals that have free access to food, our aim was to distinguish between direct and indirect effects of vitamin A on bone.

## Materials and methods

### Animals and experimental design

This study complies with the ARRIVE guidelines and was carried out in strict accordance with the UK Animals (Scientific Procedures) Act, 1986 and associated guidelines, EU Directive 2010/63/EU for animal experiments. The protocol was approved by the Committee on the Ethics of Animal Experiments of the University of Uppsala (Permit Number: C254/7). Animals and the two experimental designs have been described in detail previously (16,17). Briefly, in both studies we used male Sprague-Dawley rats, 5 weeks of age, which were obtained from Möllegaards Breeding Centre Ltd (Skensved, Denmark). They were acclimatized for 1 week and kept in groups of two or three animals, and they had free access to water. The rats were then randomly divided into a treatment and a control group. In the free access experiment there where 12 rats/group and in the pair-fed experiment 10 rats/group. In both studies they were fed a standard diet (Lactamin R36, Stockholm, Sweden) containing 12 IU vitamin A/g pellet (‘Control’), or a standard diet supplemented with 1,700 IU vitamin A/g pellet (‘Vitamin A’). The important difference between the two studies is that in the first study the animals had free access to food, and in the second study the controls were pair-fed, i.e. the control animals were fed the same amount of chow as that consumed by the vitamin A group to keep food intake the same. Vitamin A was added to the pellets in the form of retinyl palmitate and retinyl acetate. At the end of the experiment (day 7), the rats were killed by exsanguination from the abdominal aorta under Eqvitesin anesthesia (chloral hydrate 182 mg/kg, pentobarbital 41.7 g/kg), and all efforts were made to minimize suffering. Blood was left at room temperature for 30 min before centrifuging at 200*g* for 10 min to separate sera, and individual aliquots were stored at −70 °C pending biochemical analyses.

### Serum analyses

Serum analyses were performed as described previously (16,17), i.e. vitamin A was analyzed by HPLC at Vitas AS (Oslo, Norway) on samples shielded from light, and commercially available kits were utilized for measurement of serum concentrations of: N-terminal propeptide of type I collagen, PINP EIA (Immunodiagnostic Systems, Boldon, UK); C-terminal telopeptides of type I collagen (CTX-1), RatLaps (Nordic Bioscience Diagnostics, Herlev, Denmark); according to the manufacturer’s instructions.

### Peripheral quantitative computed tomography

Peripheral quantitative computed tomography (pQCT) measurements were performed as previously described (16,18). Briefly, the left femora were dissected out and cleaned. Femur lengths were measured using a slide caliper with an accuracy of 0.1 mm from the proximal to the distal end of the bone, with the ruler parallel to the bone. Subsequently the bone was covered with a piece of sterile non-woven compress and submerged in a centrifuge tube filled with phosphate-buffer saline and frozen at –18 °C until used for later pQCT analysis. An X-ray-based density measurement machine, pQCT (Stratec XCT Research SA+, Stratec Medizintechnik, Pforzheim, Germany, software version 5.50 R) at Uppsala University Hospital, Sweden, was used to measure femur dimension and density. The manufacturer-specified reference object (phantom) was used for calibration of the machine before start of measurements every day. Peripheral quantitative computed tomography scans were performed at the metaphysis (14% of bone length from the condyle) and at (mid-)diaphysis (at 50% of bone length). The voxel size was 0.07 mm, and the threshold value was set to 710 mg cm^−3^ for cortical bone (values above were defined as cortical bone); values ranging from 280 to 400 mg cm^−3^ were considered to be trabecular bone, and thresholds were set according to the manufacturer’s recommendations. Due to damage during dissection the sample size in the pair-fed study was *n* = 6 for controls and *n* = 8 for vitamin A-fed.

### Statistical analyses

Data were analyzed by Student’s *t* test or, when there were deviations from the normal distribution (Shapiro–Wilks test), with the Mann–Whitney *U* test. In every case, *P* < 0.05 was considered statistically significant.

### Ethical approval

All applicable international, national, and/or institutional guidelines for the care and use of animals were followed. All procedures performed in studies involving animals were in accordance with the ethical standards of the institution or practice at which the studies were conducted.

## Results

### Effects of a high dietary intake of vitamin A on general growth compared with either controls with free access to food or pair-fed controls

Serum concentrations of vitamin A had increased markedly after 7 days of high vitamin A intake (Table 1). Since anorexia and reduced weight gain are two known signs of hypervitaminosis A, we compared free access to food versus pair-feeding on food intake, body weight, femur length, and serum biomarkers of bone turnover. The free access group had 13% lower food intake and 15% lower body weight compared to controls (Table 1). This resulted in a general effect on bone growth as judged by the significantly reduced femur length and serum biomarkers of osteoclast activity (CTX-1) and osteoblast activity (PINP).

**Table 1.** Effects of a high dietary intake of vitamin A on serum vitamin A concentrations, food intake, body weight, femur length, and biomarkers of bone turnover compared to controls with free access to food and to pair-fed controls.

	Free access				Pair-fed			
	Control	Vitamin A	%-diff.	*P* value	Control	Vitamin A	% diff.	*P* value
Serum vitamin A (µM)	1.0 ± 0.04	1.8 ± 0.17	+69	***0.002***	1.6 ± 0.17	3.2 ± 2.5	+93	***0.001***^a^
Food intake (g/week)	102 ± 4.2	89.1 ± 28.1	–13	***0.036***	90 ± 0	90 ± 0	±0	1
Body weight (g)	124 ± 7.1	105 ± 5.5	–15	***<0.001***	125 ± 22	124 ± 18	–0.3	0.96
Femur length (mm)	25.5 ± 0.58	24.8 ± 0.71	–2.7	***0.028***	25.3 ± 1.1	25.4 ± 1.0	+0.3	0.91
Serum CTX-1 (ng/mL)	76.3 ± 28.9	48.8 ± 13.0	–36	***0.009***^a^	62.4 ± 15.4	55.1 ± 10.8	–12	0.18^a^
Serum PINP (ng/mL)	153 ± 51.2	109 ± 36.1	–28	***0.029***^a^	51.3 ± 13.2	64.6 ± 18.2	+26	0.079^a^

Results are presented as means ± SD; %-diff. is the % change of mean in vitamin A fed animals compared to controls. Free access: *n* = 12/group except for serum vitamin A analysis (*n* = 3/group). Pair-fed: *n* = 6–10/group. Statistically significant values are highlighted in bold.

^a^Data analyzed with Mann–Whitney *U* test.

CTX-1: C-terminal telopeptides of type I collagen; PINP: N-terminal propeptide of type I collagen.

### Effects of a high dietary intake of vitamin A on bone parameters compared with either controls with free access to food or pair-fed controls

The peripheral quantitative computed tomography (pQCT) results from the free access and pair-fed groups showed some striking differences. Whereas the total bone mineral content (BMC), total cross-sectional area (CSA), and periosteal circumference (PERI C) were similarly reduced in both groups in the distal metaphysis and in the diaphysis after high vitamin A intake, bone mineral density (BMD) and cortical bone parameters were significantly decreased in the free access group only (Table 2). The largest relative differences between the free access and pair-fed groups were found in cortical thickness (–24% versus –8.3% in the metaphysis and –10% versus +0.3% in the diaphysis). Consistent with the above described differences, the reduction in Polar Strength Strain Index (Polar SSI, a surrogate for a bone’s resistance to bending and torsion) (19) was more pronounced in the free access group.

**Table 2.** Effects of hypervitaminosis A on femoral bone compared to controls with free access to food and pair-fed controls, evaluated by peripheral quantitative computed tomography pQCT.

	Free access				Pair-fed			
	Control (*n* = 12)	Vitamin A (*n* = 12)	%-diff.	*P* value	Control (*n* = 6)	Vitamin A (*n* = 8)	% diff.	*P* value
Metaphysis								
Total BMC (mg/mm)	5.74 ± 0.62	3.90 ± 0.28	–32	***<0.001***	6.23 ± 0.96	4.34 ± 0.67	–30	***<0.001***
Total BMD (mg/cm^3^)	349 ± 12	331 ± 16	–5.2	***0.004***	371 ± 23	357 ± 22	–3.8	*0.28*
Total CSA (mm^2^)	16.4 ± 1.5	11.8 ± 0.98	–28	***<0.001***	16.7 ± 1.8	12.1 ± 1.5	–27	***<0.001***
Trab BMC (mg/cm^3^)	1.90 ± 0.23	1.25 ± 0.15	–34	***<0.001***	1.94 ± 0.16	1.32 ± 0.20	–32	***<0.001***
Trab BMD (mg/cm^3^)	192 ± 13	174 ± 13	–9.4	***0.004***	201 ± 11	188 ± 18	–6.5	*0.15*
Trab CSA (mm^2^)	9.87 ± 0.69	7.18 ± 0.75	–27	***<0.001***	9.63 ± 0.65	7.03 ± 0.88	–27	***<0.001***
Cort THK (mm)	0.11 ± 0.01	0.084 ± 0.02	–24	***0.010***	0.12 ± 0.03	0.11 ± 0.02	–8.3	*0.29*
PERI C (mm)	14.3 ± 0.67	12.2 ± 0.51	–15	***<0.001***	14.5 ± 0.76	12.3 ± 0.77	–15	***<0.001***
Diaphysis								
Total BMC (mg/mm)	3.78 ± 0.21	3.11 ± 0.22	–17	***<0.001***	3.64 ± 0.40	3.16 ± 0.20	–13	***0.013***
Total BMD (mg/cm^3^)	507 ± 16	497 ± 41	–2.0	*0.44*	510 ± 29	526 ± 34	+3.0	*0.39*
Total CSA (mm)	7.45 ± 0.46	6.28 ± 0.51	–16	***<0.001***	7.12 ± 0.25	6.03 ± 0.57	–15	***0.002***
Cort BMC (mg/mm)	2.76 ± 0.20	2.21 ± 0.22	–20	***<0.001***	2.69 ± 0.37	2.40 ± 0.17	–11	*0.073*
Cort BMD (mg/cm^3^)	1050 ± 20	1020 ± 28	–3.2	***0.003***	1080 ± 24	1060 ± 20	–1.8	*0.13*
Cort CSA (mm^2^)	2.62 ± 0.17	2.16 ± 0.17	–18	***<0.001***	2.50 ± 0.30	2.28 ± 0.15	–8.8	*0.090*
Cort THK (mm)	0.30 ± 0.02	0.27 ± 0.03	–10	***0.002***	0.29 ± 0.03	0.29 ± 0.02	+0.3	*0.94*
PERI C (mm)	9.67 ± 0.30	8.88 ± 0.37	–8.2	***<0.001***	9.45 ± 0.30	8.70 ± 0.41	–8.0	***0.003***
ENDO C (mm)	7.78 ± 0.29	7.18 ± 0.45	–7.7	***<0.001***	7.62 ± 0.20	6.86 ± 0.45	–10	***0.003***
Polar SSI (mm^4^)	4.01 ± 0.49	2.71 ± 0.33	–34	***<0.001***	3.84 ± 0.76	2.93 ± 0.41	–24	***0.013***
Marrow cavity (mm^2^)	4.83 ± 0.36	4.12 ± 0.52	–15	***<0.001***	4.62 ± 0.25	3.76 ± 0.50	–17	***0.002***

Results are presented as means ± SD; %-diff. is the % change of mean in vitamin A fed animals compared to controls. Statistically significant values are highlighted in bold.

BMC: bone mineral content; BMD: bone mineral density; Cort: cortical; CSA: cross-sectional area; ENDO C: endosteal circumference; PERI C: periosteal circumference; SSI: strength strain index; THK: thickness; Trab: trabecular.

## Discussion

In the present study we used young male rats and the same retinol doses as in the pivotal study by Moore (5). After 7 days the serum vitamin A concentrations were similar to those reported in vitamin A toxicity in humans (20). We found reductions in BMC, CSA, and PERI C in both the free access and pair-fed groups, whereas reductions in weight gain, femur length, serum bone biomarkers, BMD, and cortical parameters were found in the free access group only. Notably, in spite of the rapid vitamin A-induced bone thinning in both groups, there were no significant differences in cortical thickness in the pair-fed group. This is in agreement with our previous studies suggesting opposite effects of hypervitaminosis A on the periosteal and endosteal sides, i.e. increased bone resorption and decreased bone formation at the periosteal bone surface together with decreased bone resorption and increased bone formation at the endosteal surface (16,17).

Already 83 years ago, Strauss (4) reported that high doses of vitamin A induced a thinning of the long bones and an abnormally thin cortex in rats, which could explain the spontaneous fractures. These findings were confirmed by Rodahl (6) and several others (7,8). Rodahl (6) even described that on roentgenograms the cortical shadow was very often absent at both ends of the bones, and when the periost was removed postmortem, only spongy bone and no compact bone was found at the mentioned places. Based on the results of our present study, it seems reasonable to conclude that not only previously reported negative effects on the bone cortex, but also on bone length, serum biomarkers of bone turnover, and BMD in laboratory animals with hypervitaminosis A, primarily are caused by a systemic toxicity, with loss of appetite, reduced food intake, and general effects on growth. Instead, the major direct short-term effect of a high vitamin A intake in young rats is a reduction in bone diameter. In this context we find it interesting that higher maternal serum retinol levels in late pregnancy was recently shown to be associated with lower offspring total body BMC and bone area, but not BMD (21).

A limitation of this study is the small sample sizes. However, since our (16,22) and other previous studies (5,23) have shown that vitamin A has quite dramatic effects on bone, clear statistical significance could be reached even with few animals.
